# Normoxic Tumour Extracellular Vesicles Modulate the Response of Hypoxic Cancer and Stromal Cells to Doxorubicin In Vitro

**DOI:** 10.3390/ijms21175951

**Published:** 2020-08-19

**Authors:** Laura Patras, Marcel H. A. M. Fens, Pieter Vader, Arjan Barendrecht, Alina Sesarman, Manuela Banciu, Raymond Schiffelers

**Affiliations:** 1 Department of Molecular Biology and Biotechnology, Center of Systems Biology, Biodiversity and Bioresources, Faculty of Biology and Geology, Babes-Bolyai University, 400006 Cluj-Napoca, Romania; patras.laura88@yahoo.com (L.P.); alina.sesarman@ubbcluj.ro (A.S.); 2 Molecular Biology Centre, Institute for Interdisciplinary Research in Bio-Nano-Sciences, Babes-Bolyai University, 400271 Cluj-Napoca, Romania; 3 Department of Clinical Chemistry and Haematology, University Medical Center Utrecht, 3584 CX Utrecht, The Netherlands; m.h.a.m.fens@uu.nl (M.H.A.M.F.); pvader@umcutrecht.nl (P.V.); a.barendrecht@umcutrecht.nl (A.B.); r.schiffelers@umcutrecht.nl (R.S.); 4 Department of Pharmaceutics, Utrecht Institute for Pharmaceutical Sciences, Utrecht University, 3584 CG Utrecht, The Netherlands

**Keywords:** extracellular vesicles, doxorubicin, colon cancer, normoxia, hypoxia, macrophages

## Abstract

Extracellular vesicles (EV) secreted in the tumour microenvironment (TME) are emerging as major antagonists of anticancer therapies by orchestrating the therapeutic outcome through altering the behaviour of recipient cells. Recent evidence suggested that chemotherapeutic drugs could be responsible for the EV-mediated tumour–stroma crosstalk associated with cancer cell drug resistance. Here, we investigated the capacity of tumour EV (TEV) secreted by normoxic and hypoxic (1% oxygen) C26 cancer cells after doxorubicin (DOX) treatment to alter the response of naïve C26 cells and RAW 264.7 macrophages to DOX. We observed that C26 cells were less responsive to DOX treatment under normoxia compared to hypoxia, and a minimally cytotoxic DOX concentration that mounted distinct effects on cell viability was selected for TEV harvesting. Homotypic and heterotypic pretreatment of naïve hypoxic cancer and macrophage-like cells with normoxic DOX-elicited TEV rendered these cells slightly less responsive to DOX treatment. The observed effects were associated with strong hypoxia-inducible factor 1-alpha (HIF-1α) induction and B-cell lymphoma–extra-large anti-apoptotic protein (Bcl-xL)-mediated anti-apoptotic response in normoxic DOX-treated TEV donor cells, being also tightly connected to the DOX-TEV-mediated HIF-1α induction, as well as Bcl-xL levels increasing in recipient cells. Altogether, our results could open new perspectives for investigating the role of chemotherapy-elicited TEV in the colorectal cancer TME and their modulatory actions on promoting drug resistance.

## 1. Introduction

The tumour microenvironment (TME) is shaped by the complex interactions between malignant cells and stromal cells. These stromal cells include resident cells (e.g., epithelial and mesenchymal cells, resident macrophages, cancer-associated fibroblasts) or infiltrating cells (e.g., immune cells, mesenchymal stem cells, endothelial progenitor cells) [1,2,3,4]. Among all cell types belonging to the TME, tumour-associated macrophages (TAM) are considered the most abundant and essential stromal cells for tumour growth through their capacity to coordinate angiogenesis, inflammation, oxidative stress, invasion, and metastatic capacity of tumour cells [5,6,7].

Several studies, including our recent findings, underlined the involvement of these stromal cells in the modulation of cancer cell drug resistance [8,9,10,11]. We demonstrated that TAM orchestrate the response of C26 murine colon carcinoma cells to 5-fluorouracil, thereby protecting cancer cells against cytotoxic agent-induced oxidative stress [11]. Furthermore, an essential role in the crosstalk between tumour cells and TAM is played by signalling mediated by extracellular vesicles (EVs) that can further promote cancer progression and resistance to therapy [12,13,14]. It is known that EVs are a heterogeneous group of membranous structures secreted by most cells, especially under pathological or cellular stress conditions [15]. These vesicles can convey bioactive molecules (functional proteins, lipids, and nucleic acids) that can alter the behaviour of the recipient cells [15,16,17,18,19,20]. Tumour EVs (TEV) are able to shape a neoplastic TME that is favourable for cancer progression and to help define the therapeutic outcome of different cytotoxic drugs [17,21,22].

On the basis of these previous results, the aim of the present study was to investigate whether TEV released from C26 colon carcinoma cells treated with doxorubicin (DOX) could influence the response of cancer cells as well as TME cells under normoxic and hypoxic conditions [23,24]. Although DOX is a highly effective antineoplastic drug used for the treatment of advanced solid tumours, colon cancer cells are inherently resistant to this drug as a single-step exposure to clinically relevant doses of DOX was sufficient to select for multidrug-resistant cells [24]. As a cell model for TME cells, RAW 264.7 macrophage-like cells were selected due to their similarities with the phenotype of bone marrow-derived macrophages [25,26]. Moreover, to mimic different TME conditions, tumour cells as well as RAW 264.7 macrophage-like cells were cultured under normoxia (reflecting cell exposure to oxygen levels typical for the perivascular regions of solid tumours where neo-angiogenesis was triggered) or hypoxia (reflecting the tumour hypoxic or invasive front areas where macrophages mediate the initiation of neo-angiogenesis) [5].

Our data suggest that TEV derived from DOX-treated normoxic cells rendered hypoxic C26 and RAW 264.7 recipient cells more resistant to DOX treatment. To investigate whether the exerted effects were associated with the acquisition of a more drug-resistant phenotype in TEV-donor cells, we screened the resistance-associated molecular changes in these cells. Our findings revealed a strong anti-apoptotic response in normoxic C26 cells exposed to DOX.

## 2. Results

### 2.1. Under Normoxia, C26 Cells Were Less Responsive to DOX Compared to Hypoxia

**Figure 1:**
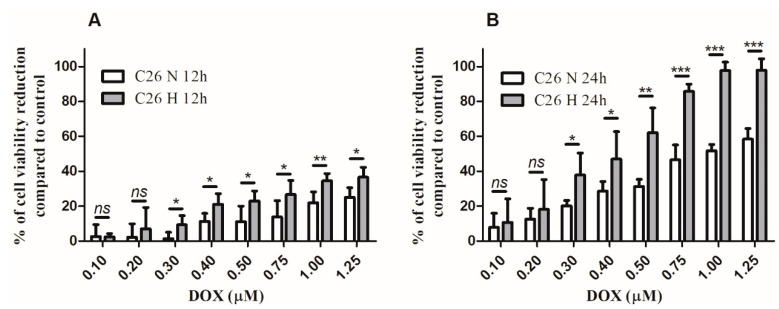
The effects of doxorubicin (DOX) on C26 murine colon carcinoma cells under normoxic and hypoxic conditions. (**A**) Percentage of cell viability reduction compared to the viability of control untreated cells after 12 h incubation of C26 cells with increasing concentrations of DOX ranging from 0.1 µM to 1.25 µM under either normoxia (C26 N 12 h) or hypoxia (C26 H 12 h). (**B**) Percentage of cell viability reduction compared to the viability of control cells after 24 h incubation of C26 cells with increasing concentrations of DOX ranging from 0.1 µM to 1.25 µM under either normoxia (C26 N 24 h) or hypoxia (C26 H 24 h). Data are shown as mean ± SD of triplicate measurements of two independent experiments; *ns*: not significant, *p* > 0.05; *, *p* < 0.05; **, *p* < 0.01; ***, *p* < 0.001.

The viability of normoxic and hypoxic C26 cells after DOX treatment was tested to determine whether the responsiveness of cancer cells could be affected by cellular stress conditions. The effects of different DOX concentrations (0.1–1.25 µM DOX) on the viability of C26 cells under normoxic and hypoxic conditions were expressed as the percentage of cell viability reduction compared to the viability of untreated control cells. These effects were assessed after 12 h and 24 h of incubation of cells with DOX and the results are shown in Figure 1A,B. IC_50_ and hypoxia cytotoxicity ratio (HCR) values for this drug under normoxia and hypoxia are shown in Table 1. These values suggested that C26 cells were about twofold less responsive to DOX under normoxic conditions at both incubation time points tested (Figure 1A,B and Table 1). On the basis of these data, the first concentration of DOX (0.3 µM) that induced statistically different cytotoxic effects on colon carcinoma cells under normoxia compared to hypoxia (12 h DOX incubation, *p* = 0.0243; 24 h DOX incubation, *p* = 0.038), at both time points of incubation, was selected throughout the experiments conducted for testing the influence of TEV on the responsiveness of these cancer cells as well as of RAW 246.7 cells to the cytotoxic drug.

### 2.2. Enhanced DOX Uptake in Hypoxic C26 Cells Compared to Its Uptake in Normoxic C26 Cells

**Figure 2:**
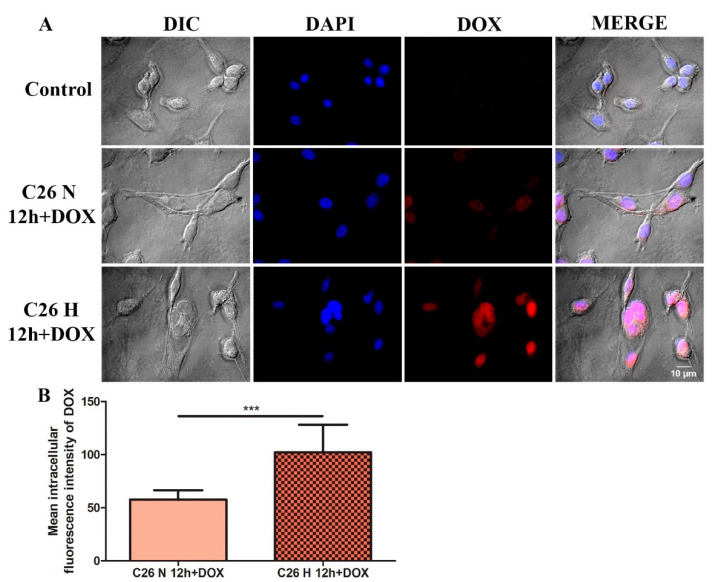
Fluorescence microscopy displaying DOX uptake pattern by C26 murine colon carcinoma cells after 12 h incubation with 0.3 µM DOX under normoxic and hypoxic conditions. (**A**) Fluorescence microscopy images acquired with different filters. DIC: differential interference contrast images of C26 cells after 12 h incubation with the drug; DAPI: fluorescence images of C26 cells subjected to 4′,6-diamidino-2-phenylindole (DAPI) staining after 12 h incubation with DOX to highlight the nuclei (excitation 365 nm, emission > 397 nm); DOX: fluorescence images of DOX uptake by C26 cells after 12 h incubation with the drug (excitation 470 nm, emission 581–679 nm); MERGE: overlays of fluorescence and DIC images. The same settings were applied for each photo taken from every experimental condition; magnification = 40×; scale bar = 10 µm; Control = untreated C26 cells cultured under normoxia. (**B**) Mean absolute intracellular DOX fluorescence was measured from several images using ImageJ software and the results were expressed as mean ± SD. Unpaired *t*-test was used for the statistical comparison between the DOX fluorescence under normoxia compared to hypoxia; ***, *p* < 0.001.

To assess whether selective cytotoxicity of DOX on C26 cells cultured under hypoxia compared to normoxia was preceded by a differential uptake of the anticancer drug in these cells, we investigated the intracellular distribution of 0.3 µM DOX after 12 h incubation by fluorescence microscopy. The results shown in Figure 2A,B show that DOX uptake by C26 cells was significantly enhanced under hypoxic conditions (by 45%, *p* = 0.0009) compared to its uptake in the same cells under normoxia (Figure 2A,B).

### 2.3. Hypoxic Conditions Stimulated the Secretion of EVs by C26 Cells

**Figure 3:**
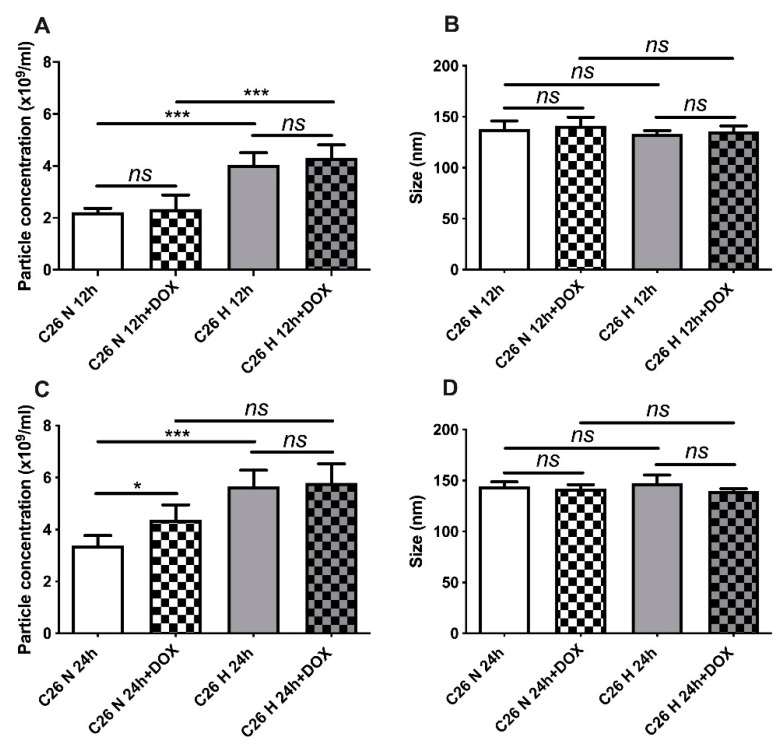
Size distribution and concentration of TEV. Nanoparticle tracking analysis was used to determine TEV concentration (**A**) and their size (**B**) under normoxia and hypoxia after 12 h incubation with 0.3 µM DOX. The production (**C**) and size (**D**) of extracellular vesicles (EVs) secreted by C26 cells after 24 h incubation with 0.3 µM DOX under normoxia and hypoxia are also shown. EV production was expressed as particle concentration/mL ± SD of triplicate measurements and data were normalised for the protein concentration obtained from cell lysates at the time of EV harvesting; *ns*: not significant, *p* > 0.05; *, *p* < 0.05; ***, *p* < 0.001.

Since cellular stress conditions are known to stimulate the release of EVs compared to physiological conditions, we investigated whether hypoxic or normoxic conditions, as well as drug treatment, could affect the number or size of EVs released by C26 cells in response to hypoxic and therapeutic stress. For this, nanoparticle tracking analysis (NTA) was used, and these data are presented in Figure 3A–D. It appears that EV production by C26 cells was enhanced by increasing cellular stress either through exposure to DOX or hypoxia, especially at the 24 h time point (Figure 3A compared to Figure 3C). Of note, hypoxia significantly increased TEV production by nearly twofold compared to their production in the normoxic C26 cells at both incubation time points tested (*p* < 0.0001, Figure 3A; *p* = 0.0489, Figure 3C). Under normoxic conditions, 0.3 µM DOX concentration enhanced the TEV production by 30% compared with untreated normoxic C26 cells (*p* = 0.0173, Figure 3A,C). Vesicle size, however, was not affected by any of the stress conditions tested (Figure 3B,D). EVs had a mean size of about 140 nm (Figure 3B,D), and likely are a mixture of exosomes and microvesicles [17].

### 2.4. TEV Effects on the Response of C26 Cells to DOX Administration

**Figure 4:**
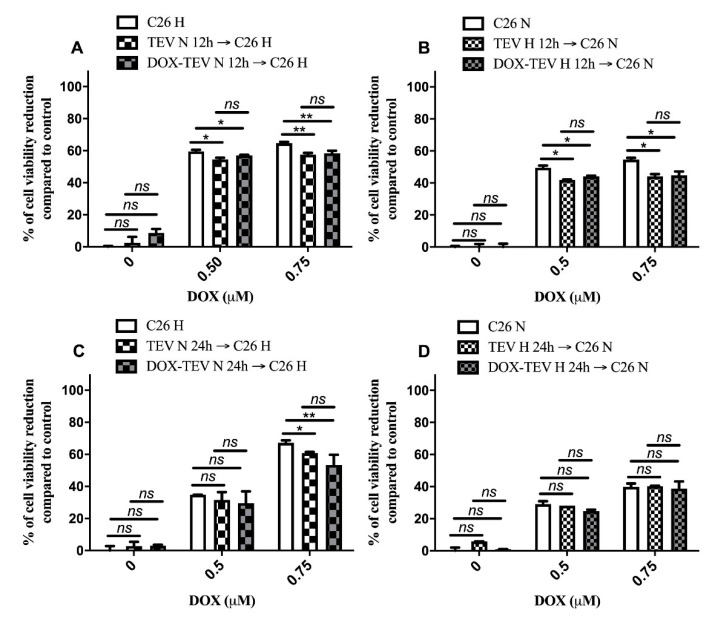
C26 cell viability reduction under normoxia and hypoxia after pretreatment with EVs from C26 cells and exposure to DOX treatment. The effects of TEV secreted by untreated normoxic C26 cells (TEV N) or by 0.3 µM DOX-treated normoxic cells (DOX-TEV N) on hypoxic C26 cells (C26 H) treated with different DOX concentrations for 12 h are shown in panel (**A**) and for 24 h are shown in panel (**C**). The effects of TEV secreted by untreated hypoxic C26 cells (TEV H) or by 0.3 µM DOX-treated hypoxic cells (DOX-TEV H) on normoxic C26 cells (C26 N) treated with different DOX concentrations for 12 h are shown in panel (**B**) and for 24 h are shown in panel (**D**). Data from interexperimental duplicates are represented as mean ± SD and expressed as a percentage compared to the corresponding controls (untreated C26 cells, C26 cells treated with 0.5 µM DOX that were not pretreated with TEV, and C26 cells treated with 0.75 µM DOX that were not pretreated with TEV, respectively); *ns*: not significant, *p* > 0.05; *, *p* < 0.05; **, *p* < 0.01.

To assess whether EVs derived from C26 cells treated with DOX could influence the response of other recipient C26 cells to the cytotoxic drug, we evaluated the viability of these cells after the pretreatment with TEV under normoxia as well as hypoxia. These results are shown in Figure 4. Our data suggest that at the earliest time point of incubation (12 h) when EVs derived from either hypoxic or normoxic C26 cells were cross-administered to recipient C26 cells, there was an improved viability of the recipient cells after drug treatment by about 10–20% (*p* < 0.05) irrespective of DOX treatment of the source cells of EVs (Figure 4A,B). It seems that EVs may inherently transport molecules and ligand moieties that cumulatively increase the survival capacity of recipient cells under stressful conditions such as DOX exposure [27,28]. Interestingly, at 24 h, only EVs derived from normoxic C26 cells enhanced the viability of the recipient hypoxic C26 cells significantly at the highest concentration of DOX compared to cancer cells that did not receive TEV pretreatments (Figure 4C,D). Thus, the viability of 0.75 µM DOX-treated hypoxic C26 cancer cells was improved by 10% (*p* < 0.05) after administration of TEV derived from untreated normoxic cells and by 20% (*p* < 0.01) after the pretreatment with TEV secreted by DOX-treated normoxic C26 cells (Figure 4C). Although the differences between the effects of TEV on the viability of DOX-treated hypoxic C26 cells are not statistically significant, it seems that normoxic DOX-elicited EV could slightly alter the response of recipient cells to DOX, in comparison with TEV obtained from untreated C26 cells.

### 2.5. TEV Effects on the Response of the RAW 264.7 Macrophage-Like Cells to DOX Administration

**Figure 5:**
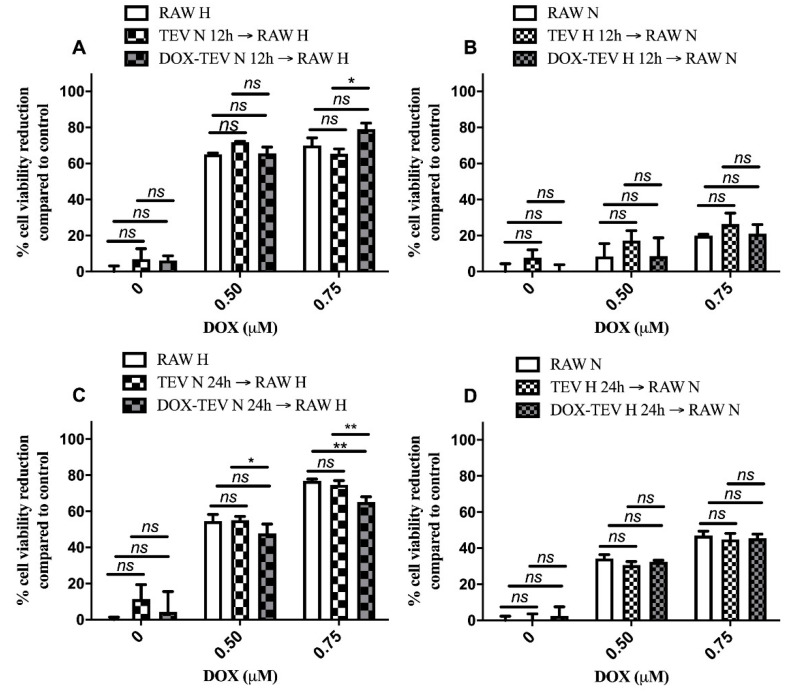
Cell viability of RAW 264.7 cells cultured under normoxia and hypoxia after pretreatment with EVs from C26 cells and exposure to DOX treatment. The effects of TEV secreted by untreated normoxic C26 cells (TEV N) or by 0.3 µM DOX-treated normoxic cells (DOX-TEV N) on hypoxic RAW 264.7 cells (RAW H) treated with different DOX concentrations for 12 h are shown in panel (**A**) and for 24 h are shown in panel (**C**). The effects of TEV secreted by untreated hypoxic C26 cells (TEV H) or by 0.3 µM DOX-treated hypoxic cells (DOX-TEV H) on normoxic RAW 264.7 cells (RAW N) treated with different DOX concentrations for 12 h are shown in panel (**B**) and for 24 h are shown in panel (**D**). Data from interexperimental duplicates are represented as mean ± SD and expressed as percentages compared to the corresponding controls (untreated RAW 264.7 cells, RAW 264.7 cells treated with 0.5 µM DOX that were not pretreated with TEV, and RAW 264.7 cells treated with 0.75 µM DOX that were not pretreated with TEV, respectively); *ns*: not significant, *p* > 0.05; *, *p* < 0.05; **, *p* < 0.01.

To investigate whether the crosstalk between cancer cells and tumour stromal cells could be affected by EVs, we added TEV produced by either hypoxic or normoxic C26 cells to DOX-treated RAW 264.7 cells. Thus, the effects of different C26 cell-derived EV pretreatments (EVs from untreated C26 cells as well as from DOX-elicited TEV) on the responsiveness of RAW 264.7 macrophages under normoxia and hypoxia were found, which are presented in Figure 5A–D. Notably, our results indicated that only TEV derived from C26 cells treated with DOX for 24 h were able to improve the viability of hypoxic RAW 264.7 cells in the presence of DOX. Thus, the viability of these cells was improved by 15% (*p* = 0.0066) compared to either control cells (no EV pretreatment) or RAW 264.7 cells pretreated with EVs from normoxic C26 cells (Figure 5C). Since the increase in RAW 264.7 cell viability occurred only after exposure to DOX-TEV isolated from normoxic C26 cells and in the presence of DOX administration (Figure 5C), this effect seems to be connected to the drug resistance induced through the transfer of specific cargo by drug-elicited TEV to the macrophages [24,29].

### 2.6. Assessment of Resistance-Associated Markers in EV Donor C26 Cells

**Figure 6:**
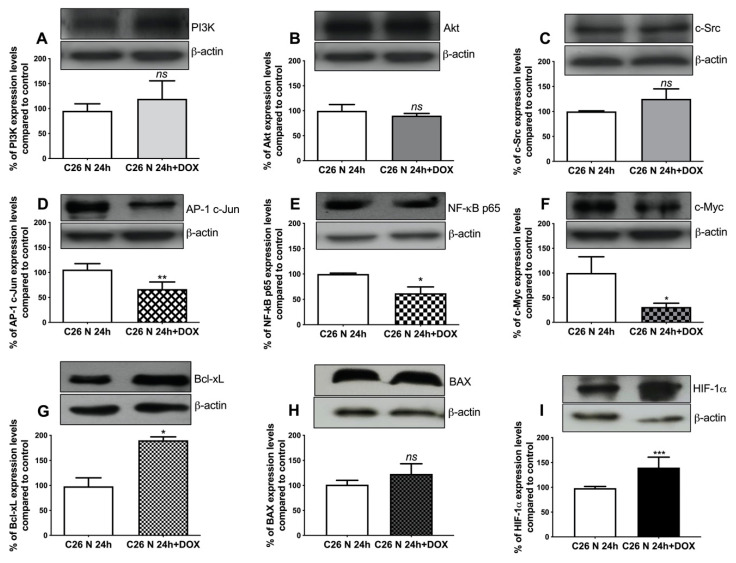
Overview on the molecular changes related to the resistance inducing mechanisms in C26 cells to DOX treatment under normoxia. Cropped Western blot images and their representative graphs displaying the percentage of protein levels after 24 h treatment with 0.3 µM DOX under normoxia compared to controls (the levels of the same proteins in untreated cell lysates) are shown for phosphatidylinositol-3 kinase (PI3K) in panel (**A**); for protein kinase B (Akt) in panel (**B**); for proto-oncogene tyrosine-protein kinase Src (c-Src) in panel (**C**); for the c-Jun subunit of AP-1 transcription factor (AP-1 c-Jun) in panel (**D**); for p65 subunit of nuclear factor kappa-light-chain-enhancer of activated B cells (NF-κB p65) in panel (**E**); for c-master regulator of cell cycle entry and proliferative metabolism (c-Myc) in panel (**F**); for B-cell lymphoma–extra-large anti-apoptotic protein (Bcl-xL) in panel (**G**), for Bcl-2-associated X protein (BAX) in panel (**H**); and for hypoxia-inducible factor 1-alpha (HIF-1α) in panel (**I**). β-actin was used as loading control. Data were expressed mean ± SD of duplicate measurements from two independent experiments. Unpaired *t*-test was used for statistical analysis of the data; *ns*: not significant, *p* > 0.05; *, *p* < 0.05; **, *p* < 0.01, ***, *p* < 0.001.

**Figure 7:**
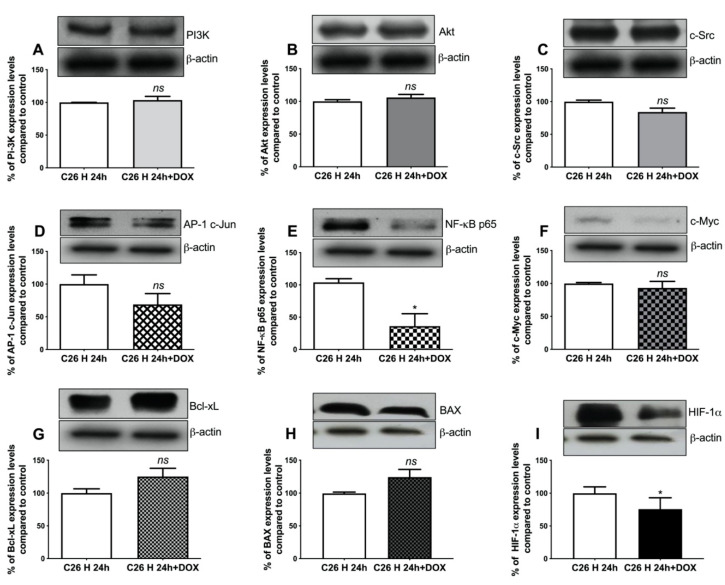
Overview on the molecular changes in hypoxic C26 cells to DOX treatment. Cropped Western blot images and their representative graphs displaying the percentage of protein levels after 24 h treatment with 0.3 µM DOX under hypoxia compared to controls (the levels of the same proteins in untreated cell lysates) are shown for PI3K in panel (**A**); for Akt in panel (**B**); for c-Src in panel (**C**); for AP-1 c-Jun (the c-Jun subunit of AP-1 transcription factor) in panel (**D**); for NF-κB p65 (the p65 subunit of the NF-κB transcription factor) in panel (**E**); for c-Myc in panel (**F**); for Bcl-xL in panel (**G**), for BAX in panel (**H**); and for HIF-1α in panel (**I**). β-actin was used as loading control. Data were expressed mean ± SD of duplicate measurements from two independent experiments. Unpaired *t*-test was used for statistical analysis of the data; *ns*: not significant, *p* > 0.05; *, *p* < 0.05.

The data presented above suggest that EVs from 24 h DOX-exposed normoxic C26 colon carcinoma cells had the strongest effects on the viability of hypoxic recipient cells. Therefore, a screening for the intratumour cell production of molecules involved in tumour-associated processes such as apoptosis (B-cell lymphoma–extra-large anti-apoptotic protein (Bcl-xL), Bcl-2-associated X protein (BAX)), proliferation (c-Jun subunit of activator protein 1 (AP-1 c-Jun), c-master regulator of cell cycle entry and proliferative metabolism (c-Myc), phosphatidylinositol-3 kinase (PI3K), protein kinase B (Akt)), inflammation (p65 subunit of nuclear factor kappa-light-chain-enhancer of activated B cells (NF-κB p65)), angiogenesis (NF-κB p65, Akt), progression and metastasis (proto-oncogene tyrosine-protein kinase Src (c-Src)), and hypoxia-inducible factor 1-alpha (HIF-1α) was performed to evaluate the DOX-induced molecular changes in normoxic versus hypoxic C26 cells, which could explain the effects of the EVs produced by these cells towards recipient cells. Our data indicated that DOX significantly affected the proliferative, inflammatory, and the angiogenic capacity of EV-donor cancer cells (Figure 6A–G and Figure 7A–G). Thus, 24 h of C26 cell DOX exposure under normoxic as well as hypoxic conditions moderately reduced (by 40–60%) the levels of a key transcription factor for tumour inflammation and angiogenesis, NF-κB p65 (*p* = 0.0276 and *p* = 0.0188, respectively) (Figure 6E and Figure 7E). Meanwhile, the intracellular production of two transcription factors associated with proliferation, AP-1 c-Jun (by 40%, *p* = 0.0059) and c-Myc (by 70%, *p* = 0.0177), was strongly reduced only under normoxic conditions (Figure 6D,F). Notably, DOX strongly induced the overexpression of the anti-apoptotic protein Bcl-xL in normoxic C26 cells exposed to the cytotoxic drug (Figure 6G) compared to hypoxia (Figure 7G), but not the levels of the pro-apoptotic protein BAX under either normoxic (Figure 6H) or hypoxic (Figure 7H) conditions. Thus, the level of this resistance-inducing marker was twice as high in DOX-treated normoxic cancer cells than in untreated C26 cells (*p* = 0.0194) and only slightly increased (by 25%) in DOX-treated hypoxic cancer cells (Figure 6G and Figure 7G). The Western blot results also indicated that HIF-1α was induced in C26 cells under hypoxic conditions compared to normoxia (Figure S7). Interestingly, the treatment of normoxic C26 cells with 0.3 µM DOX increased the levels of HIF-1α by 40% (*p* = 0.0003), while under hypoxic conditions, the same drug dose reduced the levels of this transcription factor by 25% (*p* = 0.0165) compared to control (Figure 6I and Figure 7I).

### 2.7. Assessment of Apoptotic Markers and HIF-1α in Normoxic TEV-Recipient C26 and RAW 264.7 Hypoxic Cells

**Figure 8:**
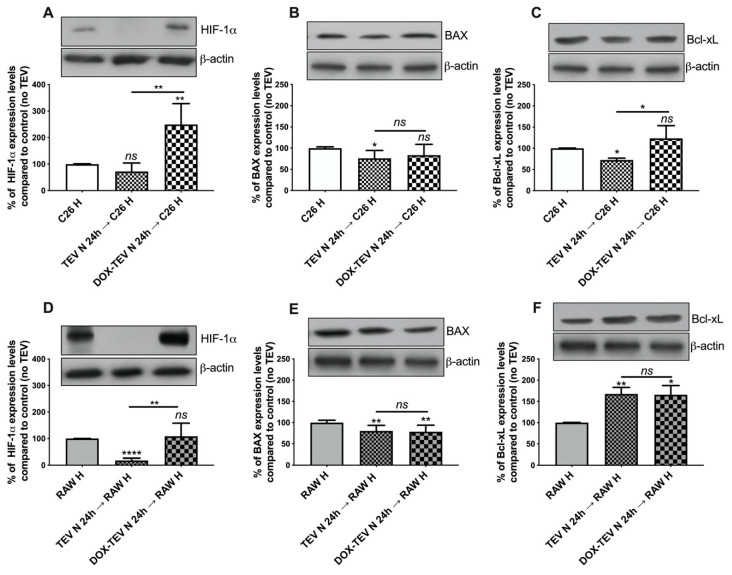
Overview on the molecular changes determined by TEV and DOX-TEV pretreatment on the recipient hypoxic C26 and RAW 264.6 cells exposed to DOX treatment. Cropped Western blot images and their representative graphs displaying the percentage of protein levels after hypoxic C26 and RAW 264.7 cell pretreatment for 24 h with C26 normoxic TEV (N TEV) or DOX-TEV (N DOX TEV) under hypoxic conditions, followed by treatment with 0.75 µM DOX under hypoxia for 24 h, as compared to controls (the levels of the same proteins in hypoxic cells treated with 0.75 µM, but no TEV pretreatment), which are shown for HIF-1α (**A**,**D**), for BAX (**B**,**E**), and for Bcl-xL (**C**,**F**). Data were expressed mean ± SD of triplicate measurements from two independent experiments. Unpaired *t*-test was used for statistical analysis of the data; *ns*: not significant, *p* > 0.05; *, *p* < 0.05, **, *p* < 0.01, ****, *p* < 0.0001.

In tight connection with the strong anti-apoptotic response in normoxic C26 cells exposed to 0.75 µM DOX, we investigated whether TEV released by these cells could induce DOX resistance in hypoxic recipient cells. Therefore, the expression levels of apoptosis-associated proteins (Bcl-xL and BAX) and HIF-1 as markers of cancer cell aggressiveness were assessed. The results presented in Figure 8 indicate that the administration of TEV altered the levels of HIF-1α in both hypoxic C26 and RAW 264.7 recipient cells (Figure 8A,D). In particular, TEV from untreated normoxic C26 cells strongly reduced by over fivefold the levels of HIF-1α in hypoxic RAW 264.7 cells (*p* < 0.0001) (Figure 8A), while normoxic DOX-TEV determined a significant increase of HIF-1α by 2.5-fold in hypoxic C26 cells (*p* = 0.009) compared to C26 hypoxic cells that received DOX treatment (Figure 8D). The Western blot results in TEV recipient cells strongly indicate that DOX-elicited TEV could strongly induce HIF-1α (*p* = 0.0058, Figure 8A; *p* = 0.0036, Figure 8D) as compared to normoxic TEV isolated from untreated C26 cells, which seem to have the opposite effect. Interestingly, DOX-elicited TEV could slightly stimulate the production of Bcl-xL in hypoxic C26 cancer cells by 25% (*p* = 0.0181, Figure 8C) compared to C26 hypoxic cells that received TEV, while RAW 264.7 recipient cells responded in a similar manner to either normoxic TEV and normoxic DOX-TEV compared to RAW 264.7 cells, which did not receive TEV (Figure 8E,F), thus showing a slight decrease by 20% (*p* = 0.0067 and *p* = 0.0091, respectively) of BAX levels in TEV recipient cells and a strong significant increase by 65% of Bcl-xL levels (*p* = 0.0043 and *p* = 0.0265, respectively).

## 3. Discussion

The EV-mediated transfer of functional molecules is part of the tumour–stroma crosstalk in the TME, modulating the therapeutic outcome and contributing to the establishment of a pro-tumourigenic setting that can assist tumour progression and could also contribute to tumour relapse after therapy and immune evasion [17,30,31]. Thus, TEV can specifically regulate the immune responses in the TME, facilitate the neoplastic vascularisation, and promote cell invasion and metastasis, as well as the formation of premetastatic niches [32,33,34,35,36]. TEV can mediate chemoresistance directly via mechanisms associated with drug removal from the cell, interference with antibody-based therapies, and transfer of molecules that ensure the malignant transformation and proliferation of recipient cells [27,37,38,39]. Indirectly, this outcome could be achieved through TEV-mediated differentiation of stromal cells into pro-tumour subsets (M2 macrophages, cancer-associated fibroblasts) involved in the acquisition of drug resistance by cancer cells [5,40,41,42]. The regulatory capacity of TEV to alter the M0/M1/M2 states has been previously described in several studies on colorectal cancer cells [43,44,45]. Moreover, it has been suggested that the outcome of TEV uptake by macrophage lineage cells on their polarisation is strongly dependent on the tumour type and specific experimental conditions used or drug treatment [44].

On the basis of this role of TEV, the current study aimed to investigate the capacity of normoxic and hypoxic DOX-induced TEV to alter the response of naïve normoxic and hypoxic C26 colon carcinoma cells and RAW 264.7 macrophages to DOX [5,46,47]. To our knowledge, the effects of chemotherapy-induced TEV homotypic and heterotypic cross-administration from hypoxic and normoxic conditions on the response of cells to treatment have not been previously investigated in these in vitro models.

Firstly, we investigated the efficacy of DOX treatment on C26 cells cultured under normoxic and hypoxic conditions in vitro in terms of viability assay. We showed that C26 cells were moderately sensitive to DOX under normoxia (Figure 1A,B) and twofold more sensitive to the drug under hypoxia (Table 1), an effect considered to be strongly dependent on the cell line used [47,48,49,50]. The decreased cell viability under hypoxic conditions might be related to the alteration of cell membrane permeability or to the decreased cytotoxic drug efflux from the cell, which would allow a higher intracellular DOX accumulation in cancer cells, as we determined in Figure 2 [48,50,51,52]. As hypoxic conditions reflect a type of cellular stress known to increase the EV secretion, our results confirmed by means of NTA analysis that TEV production was enhanced under hypoxia compared to normoxia (Figure 3A–D), which was also reported by previous findings in human colorectal cancer cells [16,53,54].

On the basis of different response patterns of C26 cells to DOX, we selected the lowest DOX concentration that exerted significant cytotoxic effects in order to further investigate the resistance-inducing mechanisms of C26 cells to this drug treatment. Thus, a modest cytotoxic drug concentration is prone to induce chemoresistance and could reflect the tumour tissue exposure to the drug in solid tumours in vivo after conventional chemotherapy due to poor tumour drug delivery, drug clearance or drug inactivation [55,56]. Additionally, it has been reported that the treatment of prostate cancer with sublethal drug doses enhanced the production of TEV carrying functional molecules, including low drug doses that could alter the phenotype of the recipient cells in vitro and could potentially be associated with drug resistance in vivo [24].

Several studies emphasised the supportive role of chemotherapy-induced TEV (in response to DOX, paclitaxel, and melphalan treatment in human prostate, breast, and myeloma cancer cells, respectively), as well as the role of both cancer and stromal cells in the TME in promoting cancer cell resistance to anticancer therapies [5,24,27,28,42,57,58]. Our results have shown that cross-administration of normoxic DOX-elicited TEV to hypoxic naïve C26 and RAW 264.7 cells for 24 h rendered recipient cells slightly less responsive to DOX treatment (Figure 4A–D and Figure 5A–D). This protective effect towards hypoxic cancer and stromal cells, as well as the DOX-enhanced EV production compared to untreated cells (Figure 3C), suggested the potential role of DOX-induced TEV in mediating chemoresistance in the TME of colorectal cancers.

To gain further insight into the protective effect of DOX-elicited TEV towards C26 and RAW 264.7 recipient cells, we investigated cytotoxic drug-induced molecular changes associated with drug resistance in EV donor cells (Figure 6A–G and Figure 7A–G). The expression levels of NF-κB p65 (Figure 6E and Figure 7E) were moderately reduced in normoxic and hypoxic C26 cells after 24 h exposure to DOX. Although this effect might seem to ensure a DOX-mediated reduction of the malignancy of C26 cells exposed to this drug, with an additional antiproliferative effect observed only under normoxic conditions via the reduction of AP-1 c-Jun and c-Myc transcription factor levels (Figure 6D,F), the capacity of DOX to induce a strong anti-apoptotic response only in normoxia-treated cells was suggested by Bcl-xL overexpression (Figure 6G). The increased levels of this anti-apoptotic protein were also consistent with the strong inhibition of c-Myc, which occurred under the same conditions, as Bcl-xL induction was reportedly involved in the blockage of cell death by apoptosis driven by c-Myc in response to antimitotic agents [59,60]. Since Bcl-xL overexpression is closely related to cancer cell survival and acquisition of a cancer cell’s invasiveness potential, it is likely that DOX-elicited TEV from cells overexpressing Bcl-xL could also convey anti-apoptotic and prometastatic signals to the recipient cells [28,61]. Consistent with a strong anti-apoptotic response and the earlier observations that normoxic DOX-TEV seem to confer a protective effect on hypoxic recipient cells, we detected that normoxic C26 cells exposed to DOX also displayed an increased level of HIF-1α protein, which further supports the acquisition of chemotherapeutic resistance in these cells [62], whereas the reduction of HIF-1α in hypoxic C26 cells after DOX treatment could suggest the sensitisation of these cells to the drug (Figure 6I and Figure 7I) [63,64].

It has been suggested for example in pancreatic cancer that TEV could promote M2 macrophage polarisation by conveying HIF-1α protein or that TEV could convey entire or cleaved Bcl-xL protein, as well as other proteins and molecules (miRNA or lncRNA) that promote the anti-apoptotic pathways after internalisation by cells, and could even lead to the production of Bcl-xL or other anti-apoptotic proteins in TEV recipient cells [44,65,66]. Thus, we further investigated whether we could detect molecular changes in the slightly less responsive hypoxic naïve C26 and RAW 264.7 cells to DOX after the pretreatment with normoxic TEV and DOX-elicited TEV (Figure 4C and Figure 5C). The results presented in Figure 8 indicate that specific molecular changes associated with the acquisition of chemoresistance after TEV administration could be responsible for the alteration of the behaviour of naïve cells. Importantly, the different degree of HIF-1α activation in both hypoxic recipient cell lines after pretreatment with DOX-TEV but not with normoxic TEV (Figure 8A,D) support the role of chemotherapy-induced TEV in driving both homotypic and heterotypic cellular responses associated with resistance to cancer therapy and cancer aggressiveness [62,67,68,69]. Moreover, the significant increase of Bcl-xL expression levels (Figure 8C) in hypoxic C26 cells pretreated with DOX-TEV compared to the TEV-only pretreatment indicates that TEV reflects the parental cell phenotype. Importantly, significant molecular changes were observed via heterotypic normoxic TEV transfer to hypoxic RAW 264.7, which triggered a strong anti-apoptotic phenotype in these cells (Figure 8E,F) coupled with a strong HIF-1α induction (Figure 8D) by DOX-TEV. The cumulative effect of TEV could therefore possibly reprogram monocyte immunophenotype towards a more immunosuppressive form, which could play a pivotal role in driving tumour aggressiveness and therapeutic resistance [69].

Altogether, these results demonstrated that homotypic and heterotypic transfer of DOX-elicited TEV from cells more resistant to the drug protected hypoxic cancer and TME cells towards the same drug treatment, emphasising the potential contribution of DOX-elicited TEV in mediating chemoresistance in the TME of colorectal cancer. This effect could be assigned to a strong anti-apoptotic response displayed by the EV donor cancer cells after DOX treatment, which could be associated with the acquisition of a more drug-resistant phenotype. Thus, our results might imply that as the drug first reaches the perivascular areas and exerts its effect on the normoxic tumour cells, it triggers the release of TEV capable of promoting drug resistance responses in recipient hypoxic tumour cells or stromal cells infiltrated in the hypoxic areas, eventually leading to the acquisition of tumour drug resistance. Nevertheless, our preliminary data open new avenues for future studies addressing TEV content and TEV-driven molecular changes in recipient cells that are needed to mechanistically demonstrate the functional role of TEV in promoting an adaptive response to drug exposure in the solid tumour milieu, with high clinical therapeutic and diagnostic potential.

## 4. Materials and Methods

### 4.1. Cell Lines and Culture Conditions

C26 murine colon carcinoma cells (Cell Line Services GmbH, Eppelheim, Germany) were cultured in Roswell Park Memorial Institute (RPMI) 1640 medium (Gibco, Breda, The Netherlands) supplemented with 2 mM L-glutamine (Gibco). RAW 264.7 murine macrophage cells (TIB-71, ATCC, Manassas, VA, USA) were maintained as a monolayer in Dulbecco’s modified Eagle’s medium (DMEM) (Gibco). RAW 264.7 cells were used as a model of macrophage-like cells as they have characteristic monocyte/macrophage morphology and reportedly mimic bone marrow-derived macrophages [25,26]. Both media were supplemented with 10% heat-inactivated foetal bovine serum (FBS) (Gibco), 100 IU/mL penicillin, and 100 μg/mL streptomycin (Gibco), and cell cultures were maintained at 37 °C in a humidified atmosphere containing 5% CO_2_ (and we will further refer to these conditions as normoxic). Hypoxic conditions were set at 1% O_2_, 5% CO_2_, and 94% N_2_ by using an in vivO_2_ 1000 Ruskinn humidified hypoxia workstation (Biotrace International, Mid Glamorgan, Baker Ruskin, UK).

### 4.2. Cell Viability Determination

To test the cytotoxicity of doxorubicin (DOX) towards C26 cells cultured in normoxic and hypoxic conditions for selecting the appropriate drug dose, we used the 3-(4,5-dimethylthiazol-2-yl)-5-(3-carboxymethoxyphenyl)-2-(4-sulfophenyl)-2H-tetrazolium inner salt (MTS) assay. For this, murine C26 colon carcinoma cells were seeded on 96-well plates (1000 cells per well) in complete pre-spun RPMI and allowed to attach overnight. Then, for the hypoxic conditions, cells were moved to hypoxia, while for normoxic conditions they were kept the same amount of time under normoxia. Afterwards, each experimental condition was subjected to DOX treatment with concentrations ranging from 0.1 to 1.25 µM DOX for 12 h and 24 h. After the appropriate incubation times in normoxic and hypoxic conditions, we replaced the medium with culture medium containing 3-(4,5-dimethylthiazol-2-yl)-5-(3-carboxymethoxyphenyl)-2-(4-sulfophenyl)-2H-tetrazolium inner salt/phenazine methosulfate (MTS/PMS) solution (Promega, Leiden, The Netherlands), according to the instructions of the manufacturer, and incubated the medium for 1 h at 37 °C. The absorbance was read at 492 nm using a SpectraMax M2e microplate reader (Molecular Devices, Wokingham, UK) and cell viability was expressed as mean percentage ± SD of cell viability reduction compared to control (untreated cells) from triplicate measurements of two independent experiments. Non-linear regression was used for calculating the IC_50_ values using dose–response curves in GraphPad Prism software version 6 for Windows (Graphpad Software, San Diego, CA, USA). Hypoxia cytotoxicity ratio (HCR) represents the ratio of IC_50_ in normoxia—IC_50_ under hypoxia and was previously used to investigate the response pattern of cells to the drug, as described by Strese et al. [70]. Thus, for example, an HCR > 1 is reportedly representative for sensitivity under hypoxia [70].

### 4.3. Fluorescence Microscopy

To assess DOX accumulation in C26 cells under either normoxia or hypoxia, we coated round-shaped cover slips with 150 µL 0.01% poly-L-lysine (Sigma-Aldrich, Steinheim, Germany) in filtered phosphate-buffered saline (PBS) and incubated them at room temperature for 1 h. Afterwards, cover slips were washed with PBS, dried for 1.5 h at 60 °C, and placed in 12-well plates in which C26 cells were seeded at a density of 5 × 10^4^ cells per well and allowed to attach overnight. The plates for the hypoxia experimental condition were moved to the hypoxia workstation with 1% oxygen and then cells from both normoxic and hypoxic conditions were incubated with 0.3 µM DOX for 12 h. Further on, C26 cells were washed twice with 300 µL PBS and fixed with 150 µL 4% paraformaldehyde for 3 h in the dark at 4 °C. The fixed cells were washed twice with PBS and then the cover slips were stained with one drop of VECTASHIELD mounting media containing 4′,6-diamidino-2-phenylindole (DAPI) (Vector Laboratories, Burlingame, CA, USA) and kept overnight at 4 °C until the fluorescence microscopy analysis. Plates were kept in the dark in both experimental conditions at all times, except for the manipulation procedures. Images were acquired using a Zeiss Axio Observer Z1 microscope (Carl Zeiss B.V., Breda, The Netherlands) equipped with Colibri LED illumination system containing a 365 nm and 470 nm LED combined with a Zeiss filterset 01 and filterset 79HE wl for measuring DAPI (>397 nm) and for DOX (581–679 nm) signal, respectively. The compounds were excited and measured separately. Then, the data were processed using the ZEN 2 Blue Edition software (Carl Zeiss Microscopy GmbH, Göttingen, Germany) and the same parameter settings were used for photos obtained from each experimental condition. Mean intracellular fluorescence intensity of DOX ± SD was evaluated using ImageJ software (National Institute of Health, Bethesda, MD, USA), and unpaired Student’s *t*-test was used to assess changes in DOX uptake by C26 cells under normoxia compared to hypoxia.

### 4.4. Preparation of Pre-Spun Media for EV Harvesting

For EV harvesting, we cultured cells from different experimental conditions in pre-spun RPMI that was obtained by ultracentrifugation of 30% FBS in culture media at 100,000× *g* and 4 °C for 16 h. Afterwards, the supernatant was filtered through 0.22 µm syringe filters (Merck Millipore, Darmstadt, Germany), diluted to 10% FBS, and enriched with penicillin/streptomycin accordingly.

### 4.5. Isolation of EVs

For harvesting C26 colon carcinoma cell-derived EVs, we seeded 1 × 10^6^ cells in complete pre-spun RPMI and allowed to attach overnight. Then, for the hypoxic conditions, the flasks were moved to hypoxia, while for normoxic conditions they were kept at the same amount of time under normoxia. Cells were treated with DOX concentrations ranging from 0.1 to 1.25 µM DOX for 12 h and 24 h. To isolate TEV, we centrifuged the cell culture supernatant collected from each experimental condition at 300× *g*, 4 °C, for 10 min to remove dead cells. The resulting supernatant was centrifuged further at 2500× *g*, 4 °C, for 10 min to remove cell debris and was then filtered through 0.45 µm syringe filters. Further on, the supernatant was transferred to ultracentrifuge tubes and subjected to ultracentrifugation at 100,000× *g* for 70 min, at 4 °C, in a fixed-angle 50.2 Ti rotor (Beckman Coulter, Pasadena, CA, USA), to pellet the EVs. Subsequently, the TEV pellet was washed once with PBS and spun again at 100,000× *g* for 70 min at 4 °C. The obtained pellet was resuspended in sterile PBS. The specific presence of EVs was verified by fluorescence-activated cell sorting (FACS) by using the method developed by Clayton et al., on the basis of the presence of CD9 tetraspanin on the surface of EVs, a common EV marker that is considerably enriched in EVs shedded by cells and constitutively enriched in EV fractions obtained from colon cancer cell lines [71,72,73].

### 4.6. Immunomagnetic Bead-Based Detection of EVs by FACS

For this bioseparation assay, we obtained immunomagnetic beads from streptavidin-coated magnetic beads (T9953 JSR Magnosphere beads, JSR Life Sciences, Sunnyvale, CA, USA), which were conjugated with biotinylated rat immunoglobulin G2a, kappa light chain (IgG2a,κ) anti-mouse CD9 antibody (clone MZ3, 130-101-961, Miltenyi Biotec, Leiden, The Netherlands) by incubation with a Binding Buffer (2x concentrated buffer containing 20 mM Tris-HCl (pH 7.4), 1 mM ethylenediaminetetraacetic acid (EDTA), 2 M NaCl, 0.1% Tween 20) for 2 h, with shaking, at room temperature, and further kept overnight at 4 °C. Then, 40,000 beads per well were seeded in a 96-well plate and washed twice with washing buffer (filtered PBS containing 1% bovine serum albumin). For the EV capturing step, the CD9-coated beads were incubated for 4 h with 100 µL of sample (previously diluted in PBS) at room temperature, with shaking. For detection, after two washing steps, the beads were incubated with rat anti-mouse CD9-AF647 antibody solution (124810, clone MZ3, BioLegend GmbH, Koblenz, Germany) for 2 h at room temperature, with shaking. After two additional washing steps, the beads were resuspended in 250 µl of PBS and subjected to FACS analysis using a FACSCanto II flow cytometer (BD Biosciences, San Jose, CA, USA). Data were analysed using the software BD FACSDiva version 8.0.1 (BD Biosciences) and expressed as mean fluorescence intensity (MFI) ± SD of duplicate measurements from two independent experiments and were corrected for controls (PBS only). The isotype control AF647 rat monoclonal IgG2a,κ (400526, clone RTK2758, BioLegend) was used for tracking non-specific binding of the primary antibody. The results of the FACS analysis confirming the presence of CD9-enriched extracellular vesicles after ultracentrifugation are presented in Figure S1.

### 4.7. Nanoparticle Tracking Analysis (NTA)

For the particle size distribution analysis of the isolated TEV from each experimental condition, we diluted the EVs pelleted by ultracentrifugation by 40-fold in sterile PBS and subjected them to triplicate measurements via NTA using a NanoSight NS500 system equipped with an LM14 405 nm violet laser unit instrument (Malvern Instruments, Worcestershire, UK). This method relies on tracking the particles in Brownian motion on the basis of their capacity to scatter the light beams, determining their size and total concentration [74]. The same settings (camera level 13, detection threshold set at 5, acquisition of three movies of 60 s, measurement at 22 °C) were applied for each experimental condition, and data analysis was performed using NTA 3.1 software (Malvern).

### 4.8. Assessment of the Effects of EVs Derived from DOX-Treated C26 Cells on the Response of C26 and RAW 264.7 Cells to DOX Treatment

To investigate whether EVs isolated from DOX-treated C26 cells could affect the response of C26 and RAW 264.7 cells to DOX treatment, we tested the viability of both cell lines after these treatments using the MTS assay. Thus, 1 × 10^6^ C26 cells per T75 flask were allowed to attach overnight in pre-spun RPMI media and the subjected to normoxic or hypoxic conditions. Afterwards, C26 cancer cells were treated with 0.3 µM DOX for 12 h and 24 h. Then, fresh pre-spun media was added to the monoculture for 24 h to allow EV-shedding by cells. Isolation and size distribution of EVs were performed as described above.

To assess the effects of EVs on the response of C26 and RAW 264.7 cells to DOX treatment, we seeded cells in 96-wells in pre-spun RPMI overnight and then they were placed under normoxic or hypoxic conditions. To test whether EVs could alter recipient cell survival and trigger an adaptive response to cytotoxic treatment, we administered EVs as pretreatment on C26 cells and RAW 264.7 macrophages in amounts approximately similar to the ones produced by these cells. Specifically, the pretreatments with 4 × 10^7^ EVs per well derived from either hypoxic or normoxic C26 cells incubated with 0.3 µM DOX for 12 h or 24 h were administered to C26 cells and RAW 264.7 cells under hypoxia and normoxia, respectively. As controls, EVs isolated from untreated C26 cells cultured under the same conditions were also administered to both cell types mentioned above. Subsequently, after 24 h incubation with the pretreatments, we incubated C26 and RAW 264.7 cells with DOX (0.5 and 0.75 µM) for 24 h, and then subjected them to MTS assay. The results were expressed as percentage of cell viability reduction compared to the corresponding control cell viability (untreated C26 or RAW 264.7 cells or C26 or RAW 264.7 cells treated with 0.5 or 0.75 µM DOX, respectively). To test whether RAW 264.7 cells could also be cultured in RPMI without altering their viability, we compared the growth of these cells in RPMI to their growth in DMEM, the media commonly used for this cell line, with no differences being noted (data not shown).

### 4.9. Assessment of the Effects of C26 TEV and DOX-TEV Pretreatments on the Levels of Proteins Involved in Hypoxia Response and Apoptosis in Hypoxic C26 and RAW 264.7 Cells Subjected to DOX Treatment

To determine whether TEV derived from untreated and DOX-treated C26 cells could mediate molecular changes in recipient cells associated with molecules involved in the acquisition of chemoresistance, we seeded 1 × 10^6^ C26 cells per T75 flask under normoxic conditions and treated them for 24 h with 0.3 µM DOX. TEV isolated from T75 flasks were administered as pretreatment on hypoxic C26 and RAW 264.7 cells seeded at the same density as TEV-producing cells (1 × 10^6^ C26 cells) and subjected to hypoxic conditions in order to ensure similar amounts of TEV used. After TEV pretreatment for 24 h, we removed the media and administered a DOX treatment with a dose of 0.75 µM for an additional 24 h.

### 4.10. Preparation of Cell Lysates

To further compare different experimental conditions, we lysed cells with r adioimmunoprecipitation assay **buffer** (RIPA) (89901, Thermo Scientific, Rockford, IL, USA) containing protease inhibitor cocktail (P8340, Sigma-Aldrich, Steinheim, Germany) and phosphatase inhibitors (Sigma-Aldrich, Steinheim, Germany). Briefly, medium was removed and cells were washed with ice-cold PBS. Then, the cells monolayers were scrapped into a 15 mL tube in ice-cold PBS and centrifuged at 300× *g* for 10 min at 4 °C. The pellet was further resuspended in ice-cold complete RIPA buffer and incubated on ice for 30 min with a short vortexing step every 10 min. Total protein was separated from cellular debris by centrifugation at 15,000× *g* for 10 min at 4 °C and the supernatant was stored at −80 °C. The concentration of total protein was determined by microBCA assay (Thermo Scientific Pierce) and the data were analysed according to the instructions of the manufacturer.

### 4.11. Western Blot Analysis

To investigate the state-of-art nature of the experimental conditions after exposure of TEV-producing cells to DOX, as well as the molecular changes in TEV recipient cells, we performed Western blot analysis from cell lysates to assess the expression levels of various proliferation, inflammation, angiogenesis, and apoptotic markers. The method is described elsewhere [11]. For each sample from TEV-producing cells, we loaded 20 µg of protein per lane, while for TEV recipient cell samples, we loaded 15 µg for all proteins, except for HIF-1 transcription factor, for which 25 µg of protein was loaded. Membranes were incubated with primary antibodies overnight at 4 °C for p65 subunit of nuclear factor kappa-light-chain-enhancer of activated B cells (NF-κB p65; mouse monoclonal IgG anti-mouse, 1:500 dilution, sc-56735, Santa Cruz Biotechnology, Santa Cruz, CA, USA), c-Jun subunit of activator protein 1 (AP-1 c-Jun; rabbit polyclonal IgG anti-mouse, 1:1000 dilution, sc-45, Santa Cruz Biotechnology), B-cell lymphoma–extra-large anti-apoptotic protein (Bcl-xL; rabbit monoclonal IgG anti-mouse, 1:500 dilution, 2764, Cell Signaling), phosphatidylinositol-3 kinase (PI3K; rabbit polyclonal IgG anti-mouse, 1:1000 dilution, 4292, Cell Signaling, Beverly, MA, USA), protein kinase B (Akt; rabbit monoclonal IgG anti-mouse, 1:1000 dilution, 4691, Cell Signaling), proto-oncogene tyrosine-protein kinase Src (c-Src; rabbit polyclonal IgG anti-mouse, 1:1000 dilution, sc-018, Santa Cruz Biotechnology), c-master regulator of cell cycle entry and proliferative metabolism (c-Myc; mouse monoclonal IgG anti-mouse, 1:500 dilution, sc-42, Santa Cruz Biotechnology), Bcl-2-associated X protein (BAX; rabbit polyclonal IgG anti-mouse, 1:500 dilution, 2772S, Cell Signalling), hypoxia-inducible factor 1-alpha (HIF-1α; rabbit polyclonal IgG anti-mouse, 1:500 dilution, ab17983, Abcam, Newcastle, UK), β-actin (rabbit polyclonal IgG anti-mouse, 1:1000 dilution, sc-130656, Santa Cruz Biotechnology), and HRP-labeled IgG goat anti-rabbit (sc-2004) or goat anti-mouse (sc-2005) secondary antibodies (1 h incubation, 1:2500 dilution, Santa Cruz Biotechnology). All antibodies were diluted in 5% non-fat dry milk (Bio-Rad Laboratories, Hercules, CA, USA) prepared in Tris-buffered saline with 0.1% Tween-20 (Honeywell Atlas Ltd., London, UK). The immunocomplexes were developed using Clarity Western ECL (Bio-Rad, 170-5061) and the blots were exposed to a Kodak X-ray film (Z358487, Eastman Kodak, Rochester, NY, USA) for about 1–5 min. Films were imaged using a ChemiDoc Touch Imaging System (Bio-Rad) and analysed using ImageJ software. Results represent mean ± SD of duplicate measurements from two independent experiments. The blots used for Western blot analysis as well as the uncropped blots are shown in Figures S2–S6.

### 4.12. Statistical Analysis

For the statistical analysis, we used GraphPad Prism software. To assess whether there were significant differences between the effects of two experimental conditions on cells, we used the unpaired *t*-test. To determine whether different experimental conditions could affect the number and the size of EVs, we used one-way ANOVA with Bonferroni correction for multiple comparisons. To compare TEV effects on the response of C26 cells as well as RAW 264.7 macrophage-like cells to DOX administration, we used two-way ANOVA with Bonferroni correction for multiple comparisons. For the calculation of the IC_50_ values, we used non-linear regression to obtain dose–response curves, from which the values were calculated. *p* < 0.05 was considered statistically significant.

## Figures and Tables

**Table 1:** Hypoxia cytotoxicity ratio (HCR) and mean values of IC_50_ of DOX after 12h and 24h incubation of C26 colon carcinoma cells with the drug under normoxia and hypoxia.

Treatment	12 h Drug Treatment			24 h Drug Treatment		
	Normoxia IC_50_	Hypoxia IC_50_	HCR (N:H)	Normoxia IC_50_	Hypoxia IC_50_	HCR (N:H)
DOX	3.704	1.922	1.93	0.929	0.407	2.28

Data are expressed as mean values from two independent experiments and are represented in micromolars. IC_50_ = the half maximal inhibitory concentration; N = normoxia; H = hypoxia; HCR = hypoxia cytotoxicity ratio (IC_50_ in normoxia/IC_50_ in hypoxia).

## Supplementary Materials

**Supplementary Materials** can be found at https://www.mdpi.com/1422-0067/21/17/5951/s1.

## Abbreviations

EV(s)	extracellular vesicle(s)
TME	tumour microenvironment
TEV	tumour-derived extracellular vesicles
DOX	doxorubicin
HIF-1α	hypoxia-inducible factor 1-alpha
Bcl-xL	B-cell lymphoma–extra-large anti-apoptotic protein
TAM	tumour-associated macrophages
HCR	hypoxia cytotoxicity ratio
IC_50_	the half maximal inhibitory concentration
DIC	differential interference contrast
DAPI	4′,6-diamidino-2-phenylindole
MFI	mean fluorescence intensity
NTA	nanoparticle tracking analysis
BAX	Bcl-2-associated X protein
AP-1 c-Jun	c-Jun subunit of activator protein 1
c-Myc	c-master regulator of cell cycle entry and proliferative metabolism
NF-κB p65	p65 subunit of nuclear factor kappa-light-chain-enhancer of activated B cells
PI3K	phosphatidylinositol-3 kinase
Akt	protein kinase B
c-Src	proto-oncogene tyrosine-protein kinase Src
RPMI	Roswell Park Memorial Institute
DMEM	Dulbecco’s modified Eagle’s medium
FBS	foetal bovine serum
MTS	3-(4,5-dimethylthiazol-2-yl)-5-(3-carboxymethoxyphenyl)-2-(4-sulfophenyl)-2H-tetrazolium inner salt)
PMS	phenazine methosulfate
PBS	phosphate-buffered saline
FACS	fluorescence-activated cell sorting
EDTA	ethylenediaminetetraacetic acid
RIPA	radioimmunoprecipitation assay buffer
