# Supplementary material for: Normoxic Tumour Extracellular Vesicles Modulate the Response of Hypoxic Cancer and Stromal Cells to Doxorubicin In Vitro
Source: Int J Mol Sci. 2020 Aug 19;21(17):5951. doi: 10.3390/ijms21175951 (PMC7503554; doi:10.3390/ijms21175951)

Supplementary figures:

A

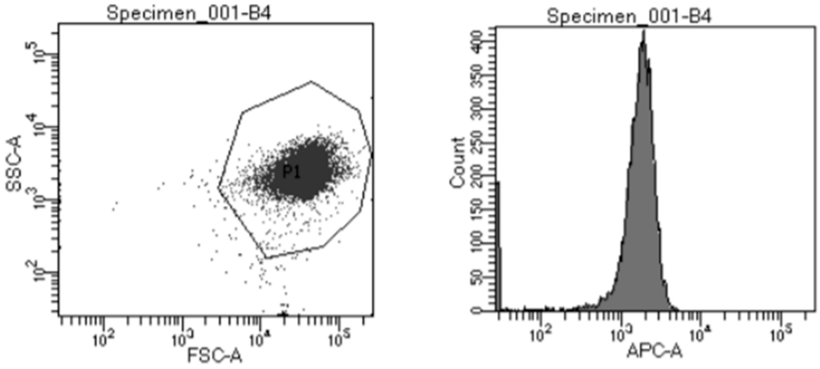

B

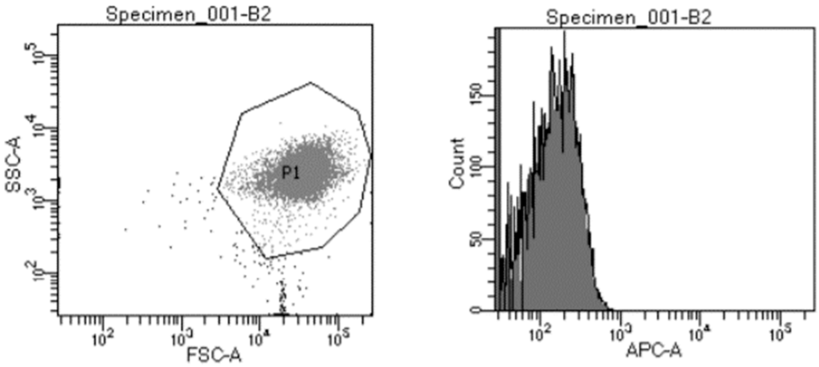

Supplementary figure S1. FACS analysis of CD9⁺ EVs isolated from C26 cells. Data shows the scatter dot-plot of beads (FSC vs SSC) with gating strategy as well as the histogram of EVs isolated with magnetic beads coated with anti-mouse CD9 antibody. CD9⁺ EVs captured with magnetic beads were stained with anti-mouse CD9 antibody (CD9-AF647, gray peak in panel **A**) or an isotype control (Panel **B**).

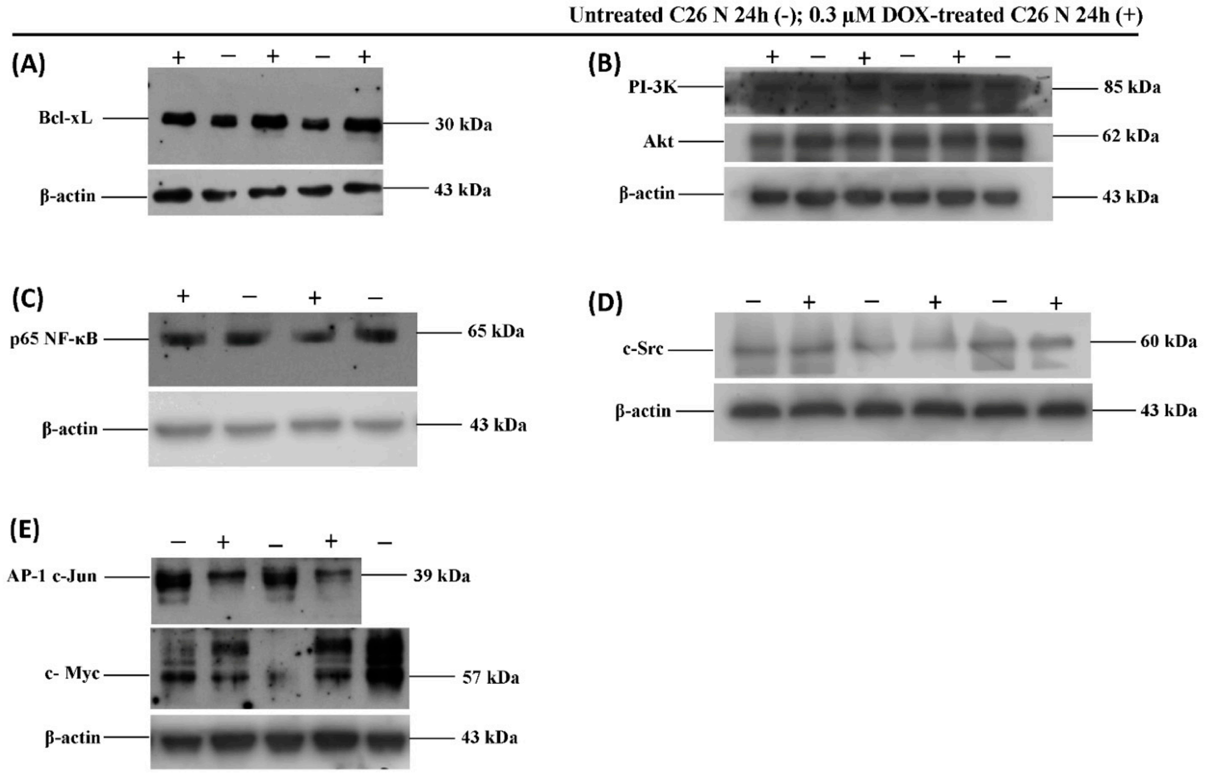

Supplementary Figure S2. All western blot images representative for the cropped western blot pictures shown in Figure 6. Each lane was loaded with a sample from a duplicate or triplicate independent experiment. β-actin was used as loading control and the proteins from each panel were probed from the same membrane as the loading control from each panel. Expression levels of Bcl-xL **(A)**, PI-3K and Akt **(B)**, p65 NF-κB **(C)**, c-Src **(D)**, AP-1 c-Jun and c-Myc **(E)** after DOX treatment were evaluated compared to the levels of the same proteins in controls (untreated cells) after normalization for β-actin.

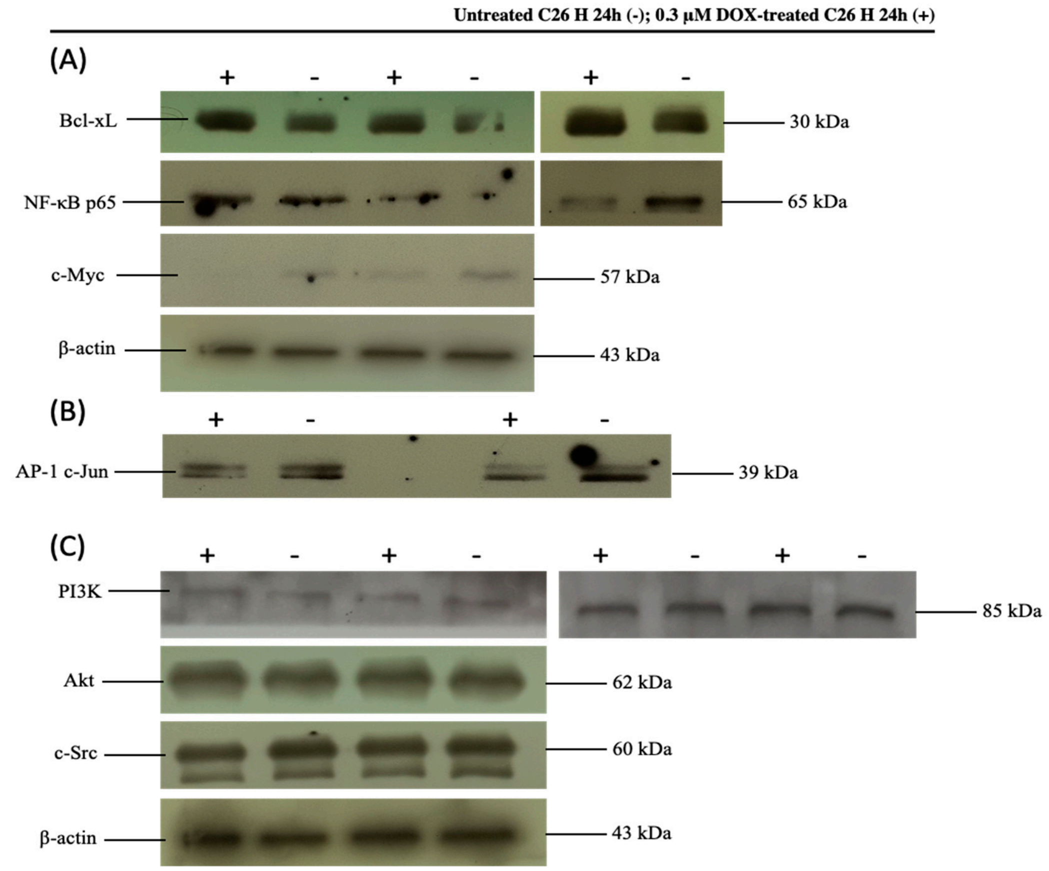

Supplementary Figure S3. All western blot images representative for the cropped western blot pictures shown in Figure 7. Each lane was loaded with a sample from a duplicate or triplicate independent experiment. β-actin was used as loading control and the proteins from each panel were probed from the same membrane as the loading control from each panel. Expression levels of Bcl-xL, p65 NF-κB, c-Myc are shown in panel **(A)**, the expression levels of AP-1 c-Jun are shown in panel **(B)**, and the expression levels of PI-3K, Akt, and c-Src are shown in panel **(C).** The expression levels of these proteins after DOX treatment were evaluated compared to the levels of the same proteins in controls (untreated cells) after normalization for β-actin.

Supplementary Figure S4. Uncropped images representative for the cropped western blot pictures shown in Figure 6. Untreated C26 cells in normoxia (-); 0.3 μM Dox-treated C26 cells in normoxia (+).

(A) PI3K

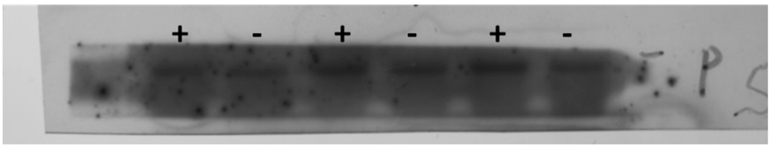

(B) Akt

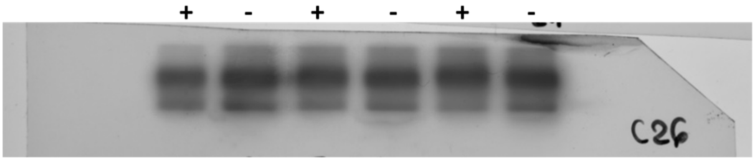

(C) c-Src

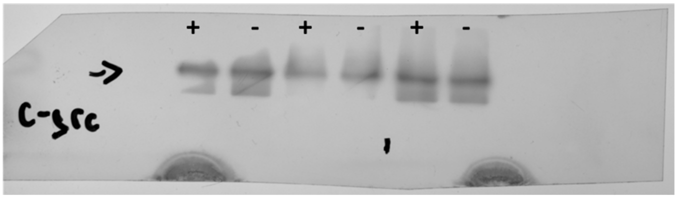

(D) AP-1 c-Jun

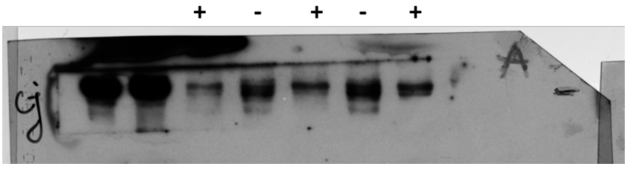

(E) NF-κB

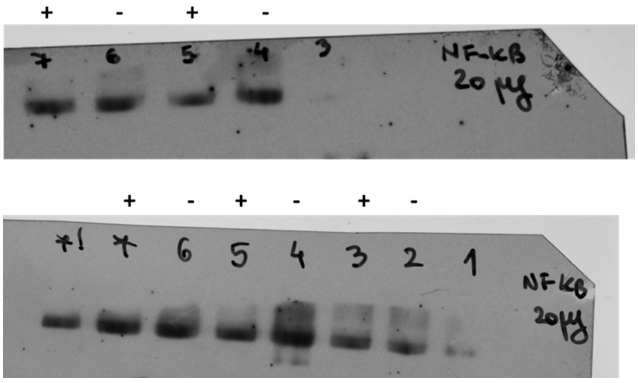

(F) c-Myc

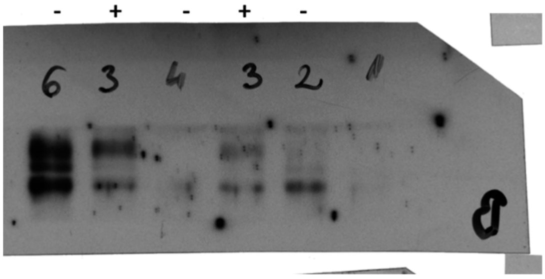

(G) Bcl-xL

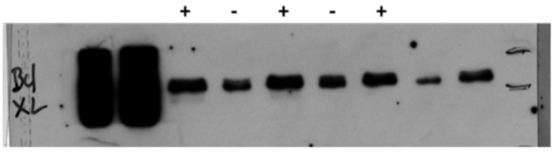

(H) β-actin (for PI3K and Akt)

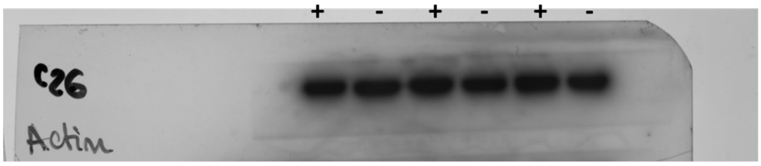

(I) β-actin (for c-Src)

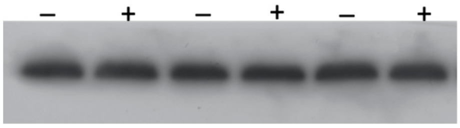

(J) β-actin (for NF-κB)

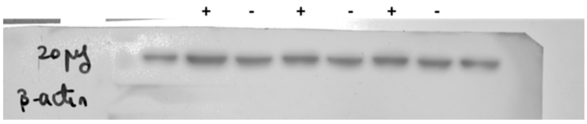

(K) β-actin (for c-Myc)

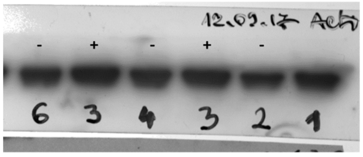

(L) β-actin (for Bcl-xL)

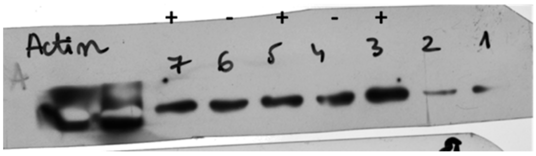

(M) BAX

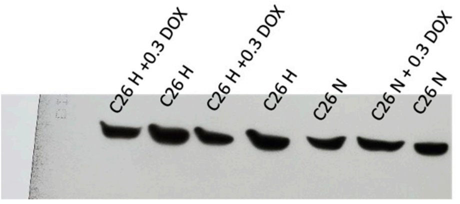

(N) HIF-1 alpha

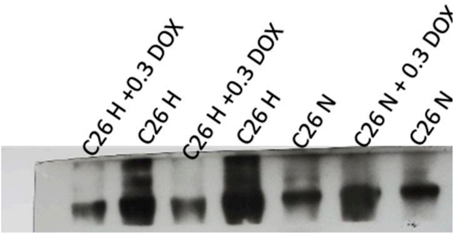

(O) β-actin for BAX, Bcl-xL and HIF-1 alpha)

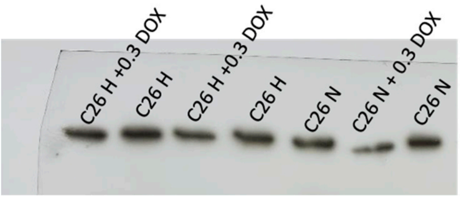

Supplementary Figure S5. Uncropped images representative for the cropped western blot pictures shown in Figure 7. Untreated C26 cells in hypoxia (**-**); 0.3 μM Dox-treated C26 cells in hypoxia (**+**).

(A) PI3K

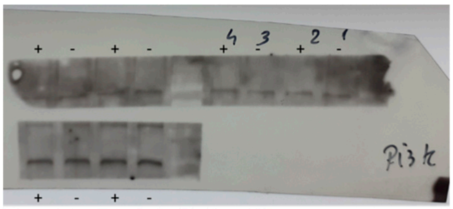

(B) Akt

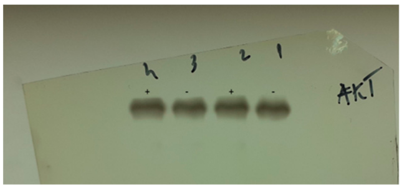

(C) c-Src

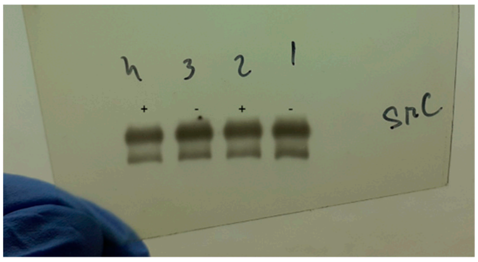

(D) AP-1 c-Jun

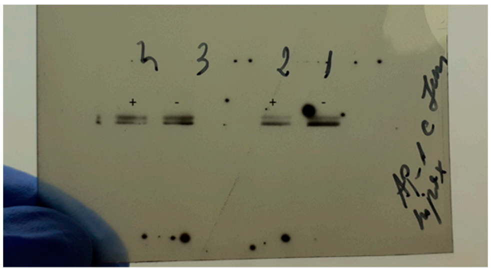

(E) NF-κB p6

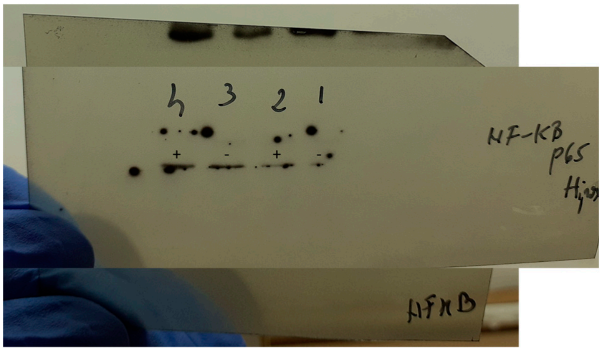

(F) c-Myc

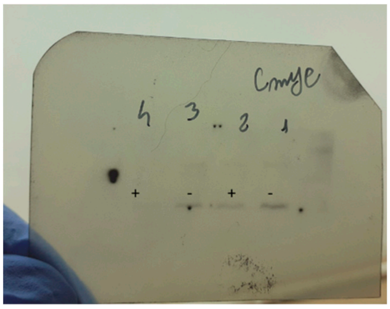

(G) Bcl-xL

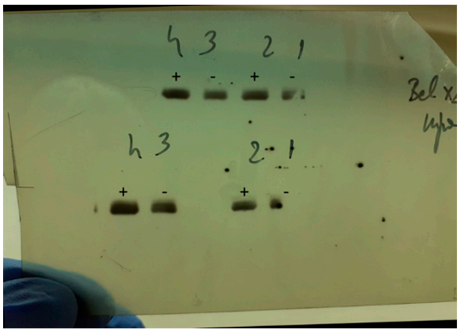

(H) β-actin (for PI3K, Akt, and c-Src)

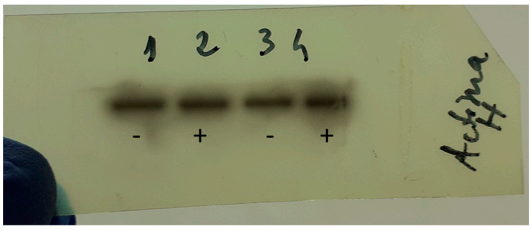

(I) β-actin (for AP-1 c-Jun, NF-κB p65, c-Myc, and Bcl-xL)

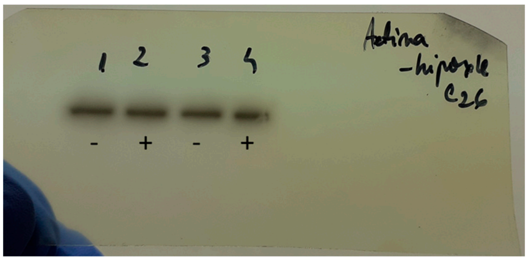

(J) BAX

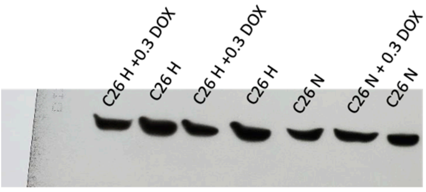

(K) HIF-1 alpha

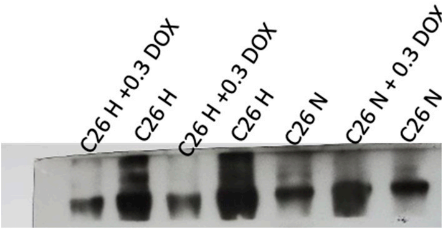

(L) β-actin for BAX, Bcl-xL and HIF-1 alpha)

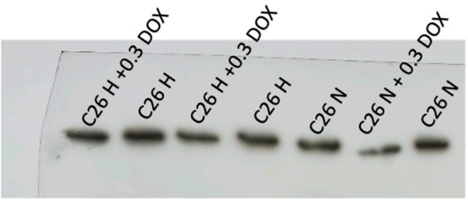

Supplementary Figure S6. Uncropped images representative for the cropped western blot pictures shown in Figure 8. Untreated C26/RAW cells in hypoxia (**C26 H/ RAW H**); 0.75 μM Dox-treated C26/RAW cells in hypoxia (**C26 H + 0.75 DOX/ RAW H + 0.75 DOX**), 0.75 μM Dox-treated C26/RAW cells in hypoxia and

pretreated with normoxic TEV (**TEV N 24h -> C26 H + 0.75 DOX, respectively TEV N 24h -> RAW H + 0.75 DOX**), 0.75 μM Dox-treated C26/RAW cells in hypoxia and pretreated with normoxic DOX-TEV (**DOX-TEV N 24h -> C26 H + 0.75 DOX, respectively DOX-TEV N 24h -> RAW H + 0.75 DOX**).

(A) HIF-1 alpha

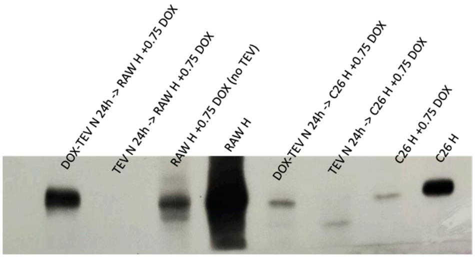

(B) Actin (for HIF-1 alpha)

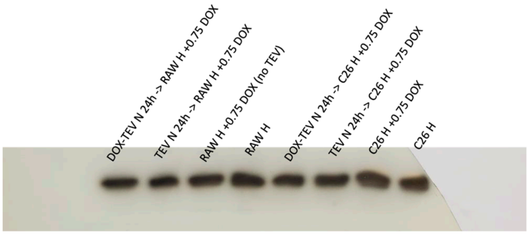

(C) Bcl-xL

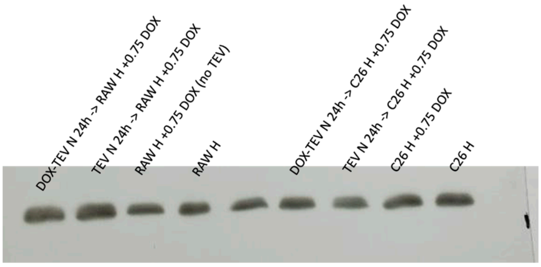

(D) BAX

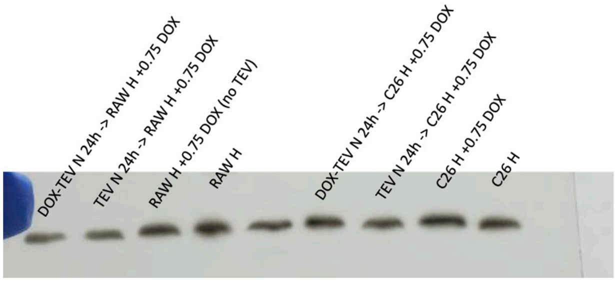

(E) Actin (for Bcl-xL and BAX)

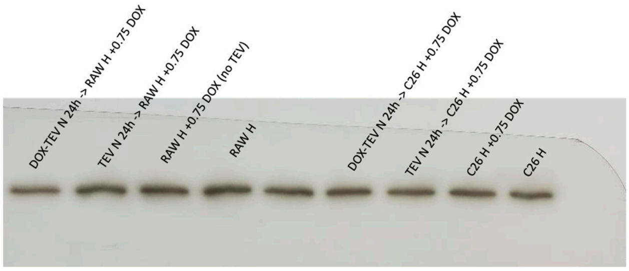

Supplementary Figure S7. HIF-1α activation in hypoxic C26 cells compared to normoxic C26 cells. Cells were treated with 0.3 μM DOX and western blot results were normalized to β-actin which was used as a loading control; *, $P<0.05$.

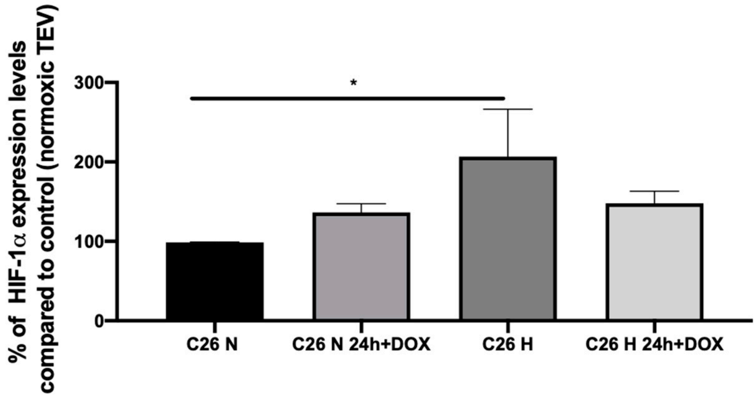

Supplement: Supplementary file 1 [file ijms-21-05951-s001.pdf]
